# Consumers’ Patient Portal Preferences and Health Literacy: A Survey Using Crowdsourcing

**DOI:** 10.2196/resprot.5122

**Published:** 2016-06-08

**Authors:** Mary Zide, Kaitlyn Caswell, Ellen Peterson, Denise R Aberle, Alex AT Bui, Corey W Arnold

**Affiliations:** ^1^ Department of Bioengineering University of California, Los Angeles Los Angeles, CA United States; ^2^ Department of Biology Florida Agricultural and Mechanical University Tallahassee, FL United States; ^3^ Medical Imaging Informatics Department of Radiological Sciences University of California, Los Angeles Los Angeles, CA United States

**Keywords:** consumer health information, health literacy, eHealth, patient portal

## Abstract

**Background:**

eHealth apps have the potential to meet the information needs of patient populations and improve health literacy rates. However, little work has been done to document perceived usability of portals and health literacy of specific topics.

**Objective:**

Our aim was to establish a baseline of lung cancer health literacy and perceived portal usability.

**Methods:**

A survey based on previously validated instruments was used to assess a baseline of patient portal usability and health literacy within the domain of lung cancer. The survey was distributed via Amazon’s Mechanical Turk to 500 participants.

**Results:**

Our results show differences in preferences and literacy by demographic cohorts, with a trend of chronically ill patients having a more positive reception of patient portals and a higher health literacy rate of lung cancer knowledge (*P*<.05).

**Conclusions:**

This article provides a baseline of usability needs and health literacy that suggests that chronically ill patients have a greater preference for patient portals and higher level of health literacy within the domain of lung cancer.

## Introduction

Life expectancy has nearly doubled in the last century [1], due in part to preventive care, screening protocols, and the conversion of previously lethal diseases into manageable, chronic conditions through medical advances. Recently, lung cancer screening with computed tomography (CT) has been shown to significantly reduce lung cancer mortality and has resulted in paradigm shifts such that screening is now a covered medical procedure by both third party payers and Medicare [2], providing the opportunity for more patients to be screened. However, lung cancer is still the single greatest cause of cancer-related death. Accurate and timely patient education is considered to be one of the critical elements underlying improvements in public health, to better inform patients of the underlying causes of their conditions, the measures they can take to mitigate risk, and the basis, and balance of benefits to risks, of certain interventions.

However, low levels of health literacy are seen as a hurdle to accessing health information [3]. The US Department of Health and Human Services defines health literacy as patients’ ability to acquire, read, and understand health information in order to make health decisions appropriate to their situation [4]. The National Center for Education Statistics maintains that health literacy is crucial for all adults to understand and improve their health, as they encounter health information in a variety of digital and hardcopy formats (eg, websites, blogs, federated search engines, magazine articles, pamphlets, prescription directions) [5].

Further study of patients’ health literacy in the subject of lung cancer is necessary to inform the design of patient education tools. Lung cancer screening protocols have recently been revised due to the findings in [2], demonstrating the efficacy of annual CT scans for high-risk individuals. Given the major policy change in lung cancer screening, it is expected that moderate patient education will be required to better understand the etiologies of lung cancer and basis for screening. eHealth apps are a potentially potent medium for the task of providing educational materials.

The term eHealth can be broadly described as the use of information technology in health care, examples of which include patient portals, personal fitness apps that run on mobile phones or personal computers, and digital consumer health educational guidelines. eHealth tools are increasingly available; however, availability does not ensure greater health literacy or increased use of these tools. The term usability refers to the ease of use and learnability of a tool, the measure of which can serve as one gauge of the effectiveness of a tool in helping the user reach their objectives [6]. Usability is necessary to ensure use of and application of the information within eHealth technologies [7,8]. Contributing to perceived usability is perceived feasibility. Feasibility, which can be defined as the effectiveness of an intervention, can be further divided into eight areas of focus, including acceptability, for example, how users react to an intervention, and demand, for example, an estimated need of a tool [8].

While no design will be able to anticipate all information needs, the content of a patient portal has the potential to influence perceived usability and satisfaction, as well as improve health literacy and outcomes [9,10]. Generally, a patient portal is a secure website that provides individuals with access to their personal medical information [11]. Although there has not been lengthy discussion on the content of portals in particular, the content of personal health records (PHRs) has received more attention. Basic consensus has been reached on certain data points that should be included: problem lists, procedures, lab tests, diagnoses, and notes [7,12-15]. These data points would likely prove beneficial in a portal as well. Portals also have a range of functions not available in PHRs, such as the ability to email a physician, renew a prescription, view clinical reports, and make appointments. However, patient information needs may differ by diagnosis, and in order to improve health literacy and outcomes, some diagnoses may require more specific additional information, educational content, and functions [7,16]. For instance, cancer patients have been shown to want access to targeted information about their particular diagnosis (benign vs cancerous), treatments (chemotherapy, radiation, surgery), and prognosis (quality of life, 5-year survival) [17]. However, the specific needs of cancer patients by diagnosis are not well known. Identifying these needs, as well as their literacy levels on specific subjects, would prove helpful in the design of tools meant to serve specific populations, such as lung cancer. Before this can be done, it is necessary to establish a baseline of a general population’s information needs.

Additionally, as lung cancer is the leading cause of cancer death and the majority of lung cancer cases are due to tobacco smoke, it is important that the general population be informed regarding lung cancer and the means through which risk can be mitigated (smoking cessation) [18]. While campaigns to inform the general population of this health issue have been wide spread, it is important to document how effective these campaigns have been in providing health consumers with knowledge, especially when considering recent reforms to screening protocol. Assessment of lung cancer literacy can help to inform portal module design aimed at improving literacy rates.

As an initial starting point, we were interested in two areas of study: (1) understanding the perceived usability and, in turn, feasibility of information content of patient portals of a convenience cohort taken from the general public, and (2) the demand for lung cancer‒specific health literacy of a convenience cohort taken from the general public. Our goal was to establish a baseline to understand how perspectives and literacy vary by basic patient (consumer) demographics.

## Methods

To assess baseline health information needs and preferences, as well as lung cancer knowledge, a survey was developed based on previously validated surveys [19,20], which were designed for the purposes of documenting patients’ health portal preferences and lung cancer knowledge. While the questions in our survey are taken from these validated tools, not all questions from the original surveys were used. Instead, in an effort to keep our survey concise and focused, we chose those questions that most closely focused on our concerned topics, perceived usability and feasibility of patient portals, and lung cancer knowledge. We chose to rely on prior validated surveys, as developing and validating a survey would require considerable additional research beyond our current scope. Our instrument consisted of three modules (see Multimedia Appendix 1). The first module captured information needs and preferences regarding patient portals posed as statements that were rated by participants on a 7-point Likert scale ranging from 1 (completely disagree) to 7 (completely agree). The second module consisted of factual statements about lung cancer and computed tomography with multiple choice answers, of which only one answer was correct. The third module contained demographic questions, based primarily on the US census [21]. Two free-text questions allowed participants to describe concerns and the effects of using a portal. This study was certified exempt by an internal review board committee at University of California, Los Angeles (UCLA).

### Recruitment

The survey was distributed via the website MTurk, a crowdsourcing Internet site devoted to human intelligence tasks [22] that has been used in prior medical informatics studies [23-26] and in the evaluation of consumer health tools [27,28]. The MTurk site was used as it has been demonstrated by those studies as a reliable method to survey a convenience sample and because it provided access to individuals across the country, as opposed to one geographical area. The survey was made available only to those MTurk participants within the United States. The survey was posted on the MTurk website for 3 weeks in May 2015. Participants were invited to complete the survey with the guarantee of compensation of US $1 per completed survey; each participant completed only one survey. The first 500 individuals to fully complete the survey were chosen as a convenience sample for purposes of analysis. We sought 250 participants age 41 and older, and 250 participants age 40 and younger. To do this, the survey was distributed twice, once as a survey available to those who were age 40 and younger, and once as a survey to those who were age 41 and older. Participants were asked to affirm their age cohort.

### Statistical Analysis

#### Power and Statistical Tests

A power analysis indicated that in order to demonstrate a 0.95 power with alpha=.025, we would require 324 participants. Survey results were analyzed using SPSS version 20 software. Statistically significant results are those with a *P* value ≤.05. Independent *t*-tests and one-way analyses of variance (ANOVAs) were used in univariate analyses to determine differences in information needs and preferences based on mean values of demographic variables. Chi-square was used in univariate analysis to identify differences in health literacy based on mean demographic variables. To determine if combinations of demographic variables can predict dependent variables, stepwise logistic regression was done using all significant variables from univariate analysis, with each model using the independent variable(s) that had significant univariate results for a particular question (*P* ≤.05). Logistic regression was used in order to make the data as parsimonious as possible, as the number of cases per group were limited. Alongside *P* values, log-odds, and prediction probabilities, Nagelkerke R^2^values were also reported. The Nagelkerke R^2^statistic is used to demonstrate how useful the independent variables are in predicting the dependent variable [29].

#### Variables

All variables can be seen in Multimedia Appendix 2. Due to sparse data, the demographic variables sex and race were collapsed into dichotomous variables (seen Multimedia Appendix 1). The variable sex included one response listed as “other.” This single response was eliminated from univariate analysis of portal preferences based on sex. For logistic regression, the 7-point Likert scales used to record patient portal responses were also dichotomized as follows: Agree (Scores 5-7) and Disagree (Score 1-4). For regression analysis, a score of 4 on Likert scale responses was included within the Disagree category, as the purpose of our analysis was to compare those who agreed to others. For lung cancer knowledge variables, binary values were coded as “0” for incorrect and “1” for correct.

## Results

Only surveys with no missing data elements were analyzed. Participants who returned surveys with missing data were contacted via email and invited to supply the missing data points. There was no consistent pattern to the types of data elements not completed. The surveys of participants who supplied the missing data were included in analysis. In total, out of the 500 surveys issued, 473 complete surveys were collected.

### User Demographics

The majority of participants were white (389/473, 82.2%) (see Table 1). Although accounting for fewer respondents, Asian (26/474, 5.4%) and black (25/473, 5.2%) participants were the second largest group and were roughly equivalent. The majority of participants had some college education, with very few (27/473, 5.7%) participants listing high school as their highest level of educational attainment. Roughly half of respondents reported an annual income of US $35,000 or less. Participants tended to spend over 11 hours a week online (349/473, 73.7%), but most had used a portal 10 times or less (216/473, 45.6%) or had never used a patient portal (174/473, 36.7%). This rate of portal use suggests that responses to the survey are both a measure of needs for those with experience using a portal, and expectations for those who have not used a portal before.

**Table 1:** Demographic results.

Demographics		n	%
**Age in years**			
	18-20	14	3
	21-30	131	27.7
	31-40	95	20.1
	41-50	139	29.4
	51-60	73	15.4
	61-70	19	4
	71-80	1	0.2
	Prefer not to answer	1	0.2
**Race**			
	White	389	82.2
	Asian	26	5.4
	American Indian	2	0.4
	Pacific Islander	2	0.4
	Black	25	5.2
	Another race	7	1.4
	Unknown or prefer not to answer	3	0.6
	Two or more races	19	4
**Education**			
	High school	27	5.7
	Some college	164	34.6
	Associate degree	63	13.3
	Bachelor’s degree	176	37.2
	Graduate degree	43	9
**Income, USD**			
	$0-35,000	237	50.1
	$36,000-50,000	95	20
	$51,000-75,000	80	16.9
	$76,000 or more	55	11.6
	Prefer not to answer	6	1.2
**Times using a portal**			
	Never	174	36.7
	1-10 times	216	45.6
	11-50 times	71	15
	51 times or more	9	1.9
	Prefer not to answer	3	0.6
**Time online, hours**			
	1-5 hours	35	7.3
	6-10 hours	87	18.3
	11 hours or more	349	73.7
	Prefer not to answer	2	0.4
**Sex**			
	Male	244	51.5
	Female	228	48.2
	Other	1	0.2

### Evaluation Outcomes

#### Influence of Participant Characteristics on Patient Portal Questions

Patient response frequencies to all survey questions can be seen in Multimedia Appendix 3 , and results to all univariate analyses can be seen in Multimedia Appendix 4. Demographic variables that significantly influenced participant perceptions of patient portal usability and feasibility are shown in Table 2. For all significant results, females rated statements about portal perceptions with higher positive responses. For example, the average Likert response to the question “Portals are not difficult to use” was 4.97 for males and 5.32 for females (*P*=.009). Females tended to have higher, more positive average Likert scores across all questions relating to portal use.

Respondents reporting a chronic illness also tended to have more positive views of portals, with higher average ratings than those without chronic illness. The average response was significantly higher for chronically ill participants for all four questions relating to portals (see Table 2). This trend was also seen across all questions relating to portal preferences.

Differences in responses significantly varied based on the number of times participants had used a patient portal. However, there was no consistent trend seen across answers. Additionally, while not statistically significant, 33% of participants reported concern about “unauthorized access” to their patient portal.

**Table 2:** Statistically significant results of univariate analysis of patient portal preferences.

Independent variable	Survey question	Eta^2^	*P*
**Sex** ^a^			
	Portals are not difficult to use.	0.014	.009
	Using a portal can make me accomplish tasks (eg, review my diagnoses and tests) quickly in managing my personal health information.	0.009	.044
**Chronic illness**			
	A portal can be useful to manage my personal health information.	0.025	.003
	Using a portal can make me accomplish tasks (eg, review my diagnoses and tests) quickly in managing my personal health information.	0.29	.001
	A personalized portal can suit my needs of managing my personal health information.	0.014	.034
	It should be easy to become skillful at using a portal.	0.027	.001
**Portal use**			
	A personalized portal can suit my needs of managing my personal health information.	0.036	.002
	Using a portal can make me accomplish tasks (eg, review my diagnoses and tests) quickly in managing my personal health information.	0.035	.002
	A portal can be useful to manage my personal health information.	0.023	.025
	Using a portal with a health encyclopedia can provide me with health care knowledge and education.	0.023	.025
	It should be easy to become skillful at using a portal.	0.030	.007

^a^Independent variable that utilized an independent *t* test; all others used ANOVAs.

#### Influence of Patient Characteristics on Lung Cancer Screening Knowledge

Questions pertaining to lung cancer and chest CT had multiple choice answers, of which one answer was correct. The chronic illness predictor was most frequently associated with correct responses (see Table 3). Those reporting a chronic illness had a higher rate of correct answers for three of the four significant results. However, those not reporting chronic illness performed better on the two questions with the outcome variable “Someone who has quit smoking…” and “Lung cancer is one of the most common cancers.”

**Table 3:** Statistically significant results of univariate analysis of demographic variables on lung cancer knowledge.

Independent variable	Survey question	*P*
**Education**		
	In the past, before the CT scan was introduced, the chance of dying due to lung cancer after diagnosis was high.	.005
**Income**		
	A change of cough pattern is a frequent sign of lung cancer.	.034
	Coughing up blood is a frequent sign of lung cancer.	.030
**Smoking habit**		
	Lung cancer is one of the most common cancers.	.036
**Hours online**		
	CT images are made with X-rays.	.016
	To complete a CT scan, subjects must undress their upper body.	.006
	Lung cancer is infectious.	.030
**Sex**		
	Coughing up blood is a frequent sign of lung cancer.	<.001
**Chronic illness**		
	CT images are made with X-rays.	.012
	To complete a CT scan, subjects must undress their upper body.	.017
	A change of cough pattern is a frequent sign of lung cancer.	.014
	Lung cancer is infectious.	<.001
**Portal use**		
	Lung cancer is infectious.	<.001

Other predictors with significant test results included time spent online, income, education, smoking habit, sex, and portal use. Time spent online had the second highest number of significant tests with three results. However, there was no consistent pattern observed within the rates of answers, with those who spent more time sometimes outperforming and sometimes underperforming those who spent less time. For the income predictor, correct answers to the question “Coughing up blood is a frequent sign of lung cancer” increased as income increased, until the level of US $76,000 or more. Those who made US $76,000 or more a year had a lower rate of correct answers than those who made less. For the smoking habit predictor, those who smoked had a slight but significantly lower rate of correct answers (78.4% versus 78.7%) for the question “Lung cancer is one of the most common cancers.” Men had a significantly higher rate of correct answers for the question “Coughing up blood is a frequent sign of lung cancer” than women (75.4% vs 73.7%). As portal use increased, correct answers to the question “Lung cancer is infectious” decreased. There was no pattern observed in response rates for the question “In the past, before the CT scan was introduced…” when stratified by the education predictor, nor for the question “A change of cough pattern is a frequent sign of lung cancer” response rates when stratified by the income predictor.

#### Logistic Regression

Stepwise logistic regression was performed for demographic variables that had statistically significant relationships as seen in Tables 2 and 3. Statistically significant results for logistic regression are seen in Table 4. Increased portal use was positively associated with agreeing with the statement “It should be easy to become skillful at using a portal,” meaning participants who used a portal more than 10 times were more likely to agree with the statement. However, prediction success was weak with 32.6% of outcomes correctly predicted. The chronic illness predictor was positively associated with having the correct answer for “A change of cough pattern is a frequent sign of lung cancer,” indicating that those with a chronic illness were more likely to choose the correct answer for the question. However, prediction was not improved in comparison to the constant model, remaining at 52.4%.

**Table 4:** Statistically significant results for logistic regression analysis.

Independent variable	Survey question	Log-odds	*P*	Nagelkerke *R*^2^	Prediction percentage correct, %
**Portal use**					
	It should be easy to become skillful at using a portal.	0.996	.016	0.024	32.6
**Chronic illness**					
	A change of cough pattern is a frequent sign of lung cancer.	0.611	.008	0.020	52.4

## Discussion

### Principal Results and Comparisons With Prior Work

In this study, a convenience cohort of the general public was asked to rate their perceptions of usability and feasibility of patient portals and knowledge of lung cancer, in order to demonstrate the demand for lung cancer knowledge and status of patient portal usability. Overall, we observed that respondents reporting chronic illness tended to have more positive opinions of patient portals and to perform better on lung cancer knowledge questions than those without a chronic condition. Chronically ill patients having a more positive opinion of eHealth is a finding that has also been observed [30,31]. Similarly, a review of literature on portals found that participant interest in patient portals varied by health status [32].

Interestingly, some studies have found that chronically ill patients may have lower health literacy [33,34]. In contrast, a study found that those with a chronic illness reported higher rates of literacy regarding test results [35]. In our work, it may be that the higher performance we observed relates to higher levels of educational attainment, as those respondents reporting chronic illness in our survey more frequently had Associate, Bachelor’s, and Master’s degrees than those reporting no chronic illness (14.6% vs 12.9%, 39.8% vs 36.8%, and 9.7% vs 8.8% respectively).

Having used a portal was associated with significant difference in opinions of patient portals. However, there was no consistent trend observed, with some statements being rated in higher agreement by those who had used portals more and other statements being rated higher by those who had used them less. It is also worth noting that for those who had never used a portal, these statements are measuring expectations of portals, while with those who have used portals, these statements are measuring experience. These results suggest that opinions about the usability of portals do not demonstrate a clear trend whether being drawn based on expectations or experience.

We also found that women reported more positive ratings of portals. While use does not equal preference, positive ratings may be influenced by the higher use of portals we observed in this survey (19.7% of women had used a portal over ten times, compared to 14.3% of men), which is consistent with higher eHealth resource use observed in women [36-38]. We also documented a statistically significant difference in correct responses for lung cancer knowledge stratified by the amount of time spent online. Others have found similar relationships between Internet use and health literacy [34], but our results showed no consistent trend and are inconclusive.

Although two logistic regression models had significant *P* value results, neither model had strong prediction success. While the low *P* values (<.05) indicate that the findings are unlikely due to chance, the prediction values, as well as the Nagelkerke *R*^2^values, indicate that these models will not perform prediction well. Further research is required to determine predictors that would predict answer cohorts to these questions successfully.

Finally, 33% of participants noted that they were “concerned about unauthorized access,” which was the most commonly chosen option (seem Multimedia Appendix 4 , Question 26). In other work, security was a concern for two thirds of health information consumers, although users of PHRs had less observed concern [39]. The overall percentage of participants concerned with security in this study was less than in [39]. However, a similar trend was observed with 32.5% of those who had used a portal more than 10 times were concerned with unauthorized access while 33.1% of those who had used a portal 10 times or less were concerned. Similarly, security has also been a concern [40]. This common theme suggests that eHealth users may associate eHealth tools with a lack of security. This concern has the potential to impact portal use, as it would likely limit satisfaction and perceived usability and, thus, feasibility.

### Limitations

There are several limitations to this study that may have influenced our results. Most importantly, we used a convenience sample of the first 500 respondents to a survey posted on MTurk. As such, the results derive from Internet users; those with less Internet experience may well have different views regarding patient portals and lung cancer health literacy than those documented here. Our respondents also had higher levels of education than seen in the US population [41], which may have influenced health literacy. Moreover, although we specifically asked respondents not to look up answers to lung cancer questions, we have no guarantee that responses were not informed by additional online queries.

An additional limitation is the potential bias that may be introduced by the use of a digital survey format. Others have found that patients who have used eHealth technology have more positive opinions of it than those who do not [42], suggesting that experience with technology can cause one to regard it more favorably. Here, completing a digital survey on eHealth may bias respondents to rate statements about patient portals more highly.

### Conclusion

This work documents a baseline for consumer information needs and health literacy within the domain of lung cancer. Our results suggest that women and those with chronic illness had more positive views of patient portals and that chronically ill patients had higher health literacy in lung cancer. Although chronically ill patients are a likely user group for patient portals, non‒chronically ill patients can also benefit from opportunities to manage and learn about their health as well as mitigate risk via portals. Given this, the results suggest a need for further lung cancer education with opportunities to use patient portals to better educate individuals who do not have a chronic illness about lung cancer. Further study is also needed in order for portals to better address the information needs of patients who are not chronically ill in order to improve perceived usability and feasibility.

This baseline can be used in future comparison studies as well as to inform portal design to improve usability and raise lung cancer health literacy with educational modules. Our future work includes designing a patient portal influenced by these results and then surveying a patient population at UCLA who will have access to the portal, to compare those survey results to this baseline, in order to identify any difference between them.

## Appendix

Multimedia Appendix 1

Multimedia Appendix 2

Multimedia Appendix 3

Multimedia Appendix 4

## Abbreviations

ANOVA: one-way analysis of variance
CT: computed tomography
PHRs: personal health records
UCLA: University of California, Los Angeles
