# Supplementary material for: Consumers’ Patient Portal Preferences and Health Literacy: A Survey Using Crowdsourcing
Source: JMIR Res Protoc. 2016 Jun 8;5(2):e104. doi: 10.2196/resprot.5122 (PMC4917738; doi:10.2196/resprot.5122)
Supplement: Multimedia Appendix 1 [file resprot_v5i2e104_app1.pdf]

Appendix 4.

Thank you for participating in this study to gather opinions about patient portals and lung cancer screening knowledge. Patient portals allow patients to view the information from their medical record online. Examples of tasks that can be done with a portal include reviewing medical reports and looking at educational information, such as health encyclopedia content. The goal of this questionnaire is to study the potential impact of patient portals, and your knowledge of lung cancer screening.

To fill out questions 1-7 of this survey, we ask that you put yourself in the role of a typical lung cancer screening patient. A typical lung cancer screening patient is age 55–77 years old. They have either: smoked one pack or more a day for 30 years, or smoked one pack a day or more for 20 years and been exposed to one or more risk factors. Risk factors include: exposure to cancer causing agents (e.g., arsenic, asbestos), a personal history of cancer, or family history of cancer. They may or may not be current smokers.

Once a patient is selected for screening, they receive a low dose CT scan of their lungs. A CT scan is taken by a machine that uses x-rays to create a set of images showing the inside of the lungs. The images are reviewed by an expert. Depending on what the expert sees in the images, the patient may be asked to return for a scan in another year, or advised to undergo additional follow-up exams, such as: additional medical imaging or a biopsy where a small amount of lung tissue is removed and studied under a microscope. While 10-20% of people who get a CT scan require additional follow-up exams, almost all of them (95%) will not have cancer.

Please answer all questions. You will not be compensated if you do not answer all questions.

For the following questions 1-7, please put yourself in the role of the lung cancer screening patient as outlined above. Please read each statement below and then select the circle of the number that most closely reflects your opinion, 7 being completely agree, and 1 being completely disagree.

1. Using a portal can make me accomplish tasks (e.g., review my diagnoses and tests) quickly in managing my personal health information. (7 completely agree, 1 completely disagree.)

☐ ☐ ☐ ☐ ☐ ☐ ☐

1 2 3 4 5 6 7

2. A portal can be useful to manage my personal health information. (7 completely agree, 1 completely disagree.)

☐ ☐ ☐ ☐ ☐ ☐ ☐

1 2 3 4 5 6 7

3. It should be easy to become skillful at using a portal. (7 completely agree, 1 completely disagree.)

☐ ☐ ☐ ☐ ☐ ☐ ☐

1 2 3 4 5 6 7

4. Portals are not difficult to use. (7 completely agree, 1 completely disagree.)

☐ ☐ ☐ ☐ ☐ ☐ ☐

1 2 3 4 5 6 7

5. A personalized portal can suit my needs of managing my personal health information. (7 completely agree, 1 completely disagree.)

☐ ☐ ☐ ☐ ☐ ☐ ☐

1 2 3 4 5 6 7

6. Using a portal can assist my face to face communication with my healthcare providers. (7 completely agree, 1 completely disagree.)

☐ ☐ ☐ ☐ ☐ ☐ ☐

1 2 3 4 5 6 7

7. Using a portal with a health encyclopedia can provide me with healthcare knowledge and education. (7 completely agree, 1 completely disagree.)

☐	☐	☐	☐	☐	☐	☐
1	2	3	4	5	6	7

For the following questions 8-18, please check the circle of the best answer. Please do not look up the answer online if you are unsure. If you are unsure, please check the circle “Don’t know”.

8. CT images are made with X-rays.

☐ Yes ☐ No ☐ Don’t know

9. To complete a CT scan, subjects must undress their upper body.

☐ Yes ☐ No ☐ Don’t know

10. In the past, before the CT scan was introduced, the chance of dying due to lung cancer after diagnosis was:

☐ Very High ☐ Somewhat High ☐ Somewhat Low ☐ Very Low

☐ Don’t know

11. Lung cancer is one of the most common cancers.

☐ Yes ☐ No ☐ Don’t know

12. A change of cough pattern is a frequent sign of lung cancer.

☐ Yes ☐ No ☐ Don’t know

13. Coughing up blood is a frequent sign of lung cancer.

☐ Yes ☐ No ☐ Don’t know

14. Lung cancer is hereditary.

☐ Yes ☐ No ☐ Don’t know

15. Lung cancer is infectious.

☐ Yes ☐ No ☐ Don’t know

16. A person can have lung cancer without complaint.

☐ Yes ☐ No ☐ Don’t know

17. Someone who has quit smoking has a higher risk of developing lung cancer than someone who has never smoked.

☐ Yes ☐ No ☐ Don't know

In the following questions 18-30, please fill in the circle that best reflects you. If you have no opinion or would like not to answer, please check the circle marked "Prefer not to answer".

18. Do you suffer from a chronic illness (e.g., diabetes, hypertension)?

☐ Yes

☐ No

☐ Prefer not to answer

19. What is your highest level of education?

☐ High School

☐ Some college

☐ Associate's degree

☐ Bachelor's degree

☐ Master's degree or beyond

☐ Other

☐ Prefer not to answer

20. What is your age group?

☐ 18-30

☐ 31-40

☐ 41-50

☐ 51-60

☐ 61-70

☐ 71 & beyond

☐ Prefer not to answer

21. What is your sex?

☐ Male

☐ Female

I use a different word to describe myself: _____________

☐ Prefer not to answer

22. What is your approximate annual income range?

☐ $0-$35,000

☐ $36,000-$50,000

☐ $51,000-$75,000

☐ $76,000 or greater

☐ Prefer not to answer

23. What race(s) best represent you? Please check all that apply.

☐ White

☐ Asian

☐ American Indian or Alaska Native

☐ Native Hawaiian or Other Pacific Islander

☐ Black or African American

☐ More than one race

☐ Unknown or not reported

☐ Prefer not to answer

24. In one week, how many hours do you spend on the Internet?

☐ 0 hours

☐ 1-5 hours

☐ 6-10 hours

☐ 11 hours or more

☐ Prefer not to answer

25. How many times have you used a patient health portal?

☐ 0 times (never)

☐ 1-10 times

☐ 11-50 times

☐ 51 times or more

☐ Prefer not to answer

26. Please check all concerns about using a portal to access your medical information that apply to you.

☐ I don't want to use a portal to access my information.

☐ Accessing information via a portal will increase my anxiety.

☐ I am concerned about unauthorized access.

☐ I am concerned about incorrect data being entered by healthcare personal.

☐ I have other concerns (please list them here) ____________________.

☐ I have no concerns.

☐ Prefer not to answer

27. Please list in a few words what concerns you might have about using a portal. If you have no concerns, please write “no concerns”.

28. Please list in a few words what effect, if any, using a portal would have on you. If it would have no effect, please write “no effect”.

29. Please enter your five digit zip code

30. Do you know anyone who has or had lung cancer?

☐ Yes

☐ No

☐ Prefer not to answer
