# Supplementary material for: Consumers’ Patient Portal Preferences and Health Literacy: A Survey Using Crowdsourcing
Source: JMIR Res Protoc. 2016 Jun 8;5(2):e104. doi: 10.2196/resprot.5122 (PMC4917738; doi:10.2196/resprot.5122)
Supplement: Multimedia Appendix 2 [file resprot_v5i2e104_app2.pdf]

Appendix 1.

Table 1. Demographic variables.

Predictors	Variable choices	Condensed values
**Age**		
	18-20	40 years and younger
	21-30	41 years and older (includes one prefer not to answer)
	31-40	
	41-50	
	51-60	
	61-70	
	71-80	
	Prefer not to answer	
**Income**		
	$0-35,000	$50,000 or less
	$36,000-50,000	$51,000 or more
	$51,000-75,000	Prefer not to answer
	$76,000 and above	
	Prefer not to answer	
**Education**		
	High school	Bachelor's degree or less
	Some college	Master's degree or more
	Associates degree	Data contained no prefer not to answer cases
	Bachelor's degree	
	Master's degree and beyond	
	Prefer not to answer	
**Sex**		
	Male	
	Female	
	I use a different word to describe myself:	All others eliminated
	Prefer not to answer	
**Races (Check all the apply)**		
	White	White
	Asian	All other races (includes prefer not to answer and unknown or not reported)
	American Indian or Alaska	
	Native Hawaiian or Other	
	Black or African American	
	More than one race	
	Unknown or not reported	
	Prefer not to answer	

Table 2. Predictor variables that are lung cancer and information need specific.

Predictors	Variable choices	Condensed values
**Portal Use**		
	Never	10 times or less (includes prefer not to answer)
	1-10 times	11 times of more
	11-50 times	
	51 times or more	
	Prefer not to answer	
**Hours Online**		
	0 hours	10 hours or less (includes prefer not to answer)
	1-5 hours	11 hours or more
	6-10 hours	
	11 hours or more	
	Prefer not to answer	
**Chronic Illness**		
	Yes	Yes
	No	No (includes prefer not to answer)
	Prefer not to answer	
**Smoking Habit**		
	Yes	Yes
	No	No (includes prefer not to answer)
	Prefer not to answer	

Table 3. Outcome variables for patient portal preferences.

Outcome	Variable choices	Binary values
**Using a portal with a health encyclopedia can provide me with healthcare knowledge and education.**		
	Likert scale<br>o 7-1	Agree (5-7)
		Disagree (1-4)
**Using a portal can assist my face to face communication with my healthcare providers.**		
	Likert scale<br>o 7-1	Agree (5-7)
		Disagree (1-4)
**A personalized portal can suit my needs of managing my personal health information.**		
	Likert scale<br>o 7-1	Agree (5-7)
		Disagree (1-4)
**Portals are not difficult to use.**		
	Likert scale<br>o 7-1	Agree (5-7)
		Disagree (1-4)
**It should be easy to become skillful at using a portal.**		
	Likert scale<br>o 7-1	Agree (5-7)
		Disagree (1-4)
**A portal can be useful to manage my personal health information.**		
	Likert scale<br>o 7-1	Agree (5-7)
		Disagree (1-4)
**Using a portal can make me accomplish tasks (e.g., review my diagnoses and tests) quickly in managing my personal health information.**		
	Likert scale<br>o 7-1	Agree (5-7)
		Disagree (1-4)

Table 4. Outcome variables for lung cancer screening.

Outcome	Variable choices	Binary values
**In the past, before the CT scan was introduced, the chance of dying due to lung cancer after diagnosis was:**		
	High	Correct (high)
	Somewhat high	Incorrect (all others)
	Somewhat low	
	Low	
**A change of cough pattern is a frequent sign of lung cancer.**		
	Yes	Correct (yes)
	No	Incorrect (all others)
	Don’t know	
**Someone who has quit smoking has a higher risk of developing lung cancer than someone who has never smoked.**		
	Yes	Correct (yes)
	No	Incorrect (all others)
	Don’t know	
**A person can have lung cancer without complaint.**		
	Yes	Correct (yes)
	No	Incorrect (all others)
	Don’t know	
**Lung cancer is infectious.**		
	Yes	Correct (no)
	No	Incorrect (all others)
	Don’t know	
**Lung cancer is hereditary.**		
	Yes	Correct (no)
	No	Incorrect (all others)
	Don’t know	
**Coughing up blood is a frequent sign of lung cancer.**		
	Yes	Correct (yes)
	No	Incorrect (all others)
	Don’t know	
**Lung cancer is one of the most common cancers.**		
	Yes	Correct (yes)
	No	Incorrect (all others)
	Don’t know	
**To complete a CT scan, subjects must undress their upper body.**		
	Yes	Correct (no)
	No	Incorrect (all others)
	Don’t know	
**CT images are made with X-rays.**		
	Yes	Correct (yes)
	No	Incorrect (all others)
	Don’t know	
