# Supplementary material for: Consumers’ Patient Portal Preferences and Health Literacy: A Survey Using Crowdsourcing
Source: JMIR Res Protoc. 2016 Jun 8;5(2):e104. doi: 10.2196/resprot.5122 (PMC4917738; doi:10.2196/resprot.5122)
Supplement: Multimedia Appendix 3 [file resprot_v5i2e104_app3.pdf]

Appendix 2.

Table 1. Outcome frequencies for patient portal preferences.

Outcome	1	2	3	4	5	6	7	Total
**Using a portal with a health encyclopedia can provide me with healthcare knowledge and education.**	5	7	16	40	104	125	176	473
**Using a portal can assist my face to face communication with my healthcare providers.**	6	12	27	63	120	117	128	473
**A personalized portal can suit my needs of managing my personal health information.**	1	9	11	21	93	147	191	473
**Portals are not difficult to use.**	9	17	39	85	109	112	102	473
**It should be easy to become skillful at using a portal.**	4	5	27	52	107	117	161	473
**A portal can be useful to manage my personal health information.**	2	5	5	23	73	131	234	473
**Using a portal can make me accomplish tasks (e.g., review my diagnoses and tests) quickly in managing my personal health.**	2	4	8	34	109	141	175	473

Table 2. Outcome frequencies for lung cancer screening.

Outcome	Yes	No	I don’t know	Total
**Someone who has quit smoking has a higher risk of developing lung cancer than someone who has never smoked.**	363	60	50	473
**A change of cough pattern is a frequent sign of lung cancer.**	248	55	170	473
**A person can have lung cancer without complaint.**	302	50	121	473
**Lung cancer is infectious.**	13	438	22	473
**Lung cancer is hereditary.**	97	239	137	473
**Coughing up blood is a frequent sign of lung cancer.**	352	20	101	473
**Lung cancer is one of the most common cancers.**	378	42	53	473
**To complete a CT scan, subjects must undress their upper body.**	141	202	130	473
**CT images are made with X-rays.**	282	102	89	473

	Very High	Some-what High	Some-what Low	Low	I don’t know	Total
**In the past, before the CT scan was introduced, the chance of dying due to lung cancer after diagnosis was:**	182	186	17	6	82	473
