# Supplementary material for: Consumers’ Patient Portal Preferences and Health Literacy: A Survey Using Crowdsourcing
Source: JMIR Res Protoc. 2016 Jun 8;5(2):e104. doi: 10.2196/resprot.5122 (PMC4917738; doi:10.2196/resprot.5122)
Supplement: Multimedia Appendix 4 [file resprot_v5i2e104_app4.pdf]

Appendix 3.

Table 1. All results for portal preference questions univariate analysis: A. It should be easy to become skillful at using a portal. B. Using a portal can assist my face to face communication with my healthcare providers. C. Using a portal with a health encyclopedia can provide me with healthcare knowledge and education. D. A portal can be useful to manage my personal health information. E. Portals are not difficult to use. F. Using a portal can make me accomplish tasks (e.g., review my diagnoses and tests) quickly in managing my personal health information. G. A personalized portal can suit my needs of managing my personal health information.

		A	B	C	D	E	F	G
**Age**								
	F score	1.358	1.662	1.222	1.150	0.574	1.312	1.346
	P	0.221	0.116	0.289	0.330	0.777	0.243	0.227
**Education**								
	F score	0.277	1.679	1.173	0.919	0.299	1.094	1.561
	P	0.893	0.154	0.322	0.453	0.878	0.359	0.327
**Income**								
	F score	0.950	0.771	1.424	0.278	0.682	0.567	0.531
	P	0.440	0.544	0.225	0.892	0.604	0.687	0.713
**Smoking Habit**								
	F score	0.914	1.099	1.117	1.875	0.801	0.519	1.483
	P	0.402	0.334	0.328	0.155	0.450	0.596	0.228
**Hours Online**								
	F score	1.217	0.191	1.678	1.009	0.288	2.349	1.876
	P	0.303	0.903	0.171	0.388	0.834	0.072	0.133
**Sex**								
	F score	2.277	3.089	0.442	0.293	0.339	1.718	0.001
	P	0.108	0.108	0.172	0.136	*0.009*	*0.044*	0.172
**Chronic Illness**								
	F score	6.622	0.133	0.701	6.026	0.523	7.053	3.413
	P	*0.001*	0.876	0.496	*0.003*	0.593	*0.001*	*0.034*
**Race**								
	F score	0.599	1.016	0.708	-0.173	0.381	0.508	0.187
	P	0.550	0.310	0.479	0.863	0.704	0.612	0.852
**Portal Use**								
	F score	3.600	0.852	2.803	2.803	2.203	4.252	4.317
	P	*0.007*	0.493	*0.025*	*0.025*	0.068	*0.002*	*0.002*

Table 2. All Chi-square results for lung cancer screening knowledge questions univariate analysis: A. CT images are made with X-rays. B. To complete a CT scan, subjects must undress their upper body. C. In the past, before the CT scan was introduced, the chance of dying due to lung cancer after diagnosis was: D. Lung cancer is one of the most common cancers. E. A change of cough pattern is a frequent sign of lung cancer. F. Coughing up blood is a frequent sign of lung cancer. G. Lung cancer is hereditary. H. Lung cancer is infectious. I. A person can have lung cancer without complaint. J. Someone who has quit smoking has a higher risk of developing lung cancer than someone who has not smoked.

		A	B	C	D	E	F	G	H	I	J
**Age**											
	X^2	16.57	20.39	40.25	5.71	11.21	10.92	23.20	9.71	15.74	9.93
	P	0.280	0.118	0.063	0.973	0.669	0.692	0.057	0.783	0.329	0.767
**Education**											
	X^2	6.15	6.63	34.58	12.26	8.73	2.61	10.32	7.25	13.50	12.50
	P	0.630	0.576	*0.005*	0.140	0.365	0.956	0.243	0.510	0.096	0.130
**Income**											
	X^2	6.39	8.18	16.22	9.90	16.63	17.00	8.09	11.00	11.10	11.01
	P	0.603	0.416	0.437	0.272	*0.034*	*0.030*	0.425	0.202	0.196	0.201
**Smoking Habit**											
	X^2	0.91	4.25	13.97	10.30	3.00	3.94	1.84	3.33	4.16	3.49
	P	0.922	0.373	0.082	*0.036*	0.557	0.414	0.764	0.503	0.384	0.479
**Hours Online**											
	X^2	15.68	18.01	11.70	0.97	7.45	3.99	10.35	13.93	6.45	4.32
	P	*0.016*	*0.006*	0.469	0.986	0.281	0.677	0.110	*0.030*	0.375	0.633
**Sex**											
	X^2	3.36	1.98	11.19	7.79	4.62	23.43	4.60	6.23	1.03	2.72
	P	0.499	0.739	0.191	0.099	0.328	*0.000*	0.331	0.182	0.904	0.605
**Chronic Illness**											
	X^2	12.82	12.05	9.98	1.37	12.42	0.85	4.54	56.55	6.38	7.67
	P	*0.012*	*0.017*	0.266	0.849	*0.014*	0.930	0.338	*0.000*	0.172	0.104
**Race**											
	X^2	30.13	29.68	35.39	33.23	35.33	38.54	35.59	21.94	35.31	29.26
	P	0.657	0.679	1.000	0.505	0.405	0.272	0.393	0.945	0.406	0.699
**Portal Use**											
	X^2	9.93	10.92	9.90	4.22	5.37	6.14	9.95	34.80	8.38	10.16
	P	0.269	0.206	0.871	0.837	0.717	0.631	0.268	*0.000*	0.396	0.254
